# Field pathogenomics reveals the emergence of a diverse wheat yellow rust population

**DOI:** 10.1186/s13059-015-0590-8

**Published:** 2015-02-25

**Authors:** Amelia Hubbard, Clare M Lewis, Kentaro Yoshida, Ricardo H Ramirez-Gonzalez, Claude de Vallavieille-Pope, Jane Thomas, Sophien Kamoun, Rosemary Bayles, Cristobal Uauy, Diane GO Saunders

**Affiliations:** National Institute of Agricultural Botany, Cambridge, CB3 0LE UK; John Innes Centre, Norwich Research Park, Norwich, NR4 7UH UK; The Sainsbury Laboratory, Norwich Research Park, Norwich, NR4 7UH UK; The Genome Analysis Centre, Norwich Research Park, Norwich, NR4 7UH UK; INRA, UMR BIOGER CPP, F-78850 Thiverval-Grignon, France

## Abstract

**Background:**

Emerging and re-emerging pathogens imperil public health and global food security. Responding to these threats requires improved surveillance and diagnostic systems. Despite their potential, genomic tools have not been readily applied to emerging or re-emerging plant pathogens such as the wheat yellow (stripe) rust pathogen *Puccinia striiformis* f. sp. *tritici* (PST). This is due largely to the obligate parasitic nature of PST, as culturing PST isolates for DNA extraction remains slow and tedious.

**Results:**

To counteract the limitations associated with culturing PST, we developed and applied a field pathogenomics approach by transcriptome sequencing infected wheat leaves collected from the field in 2013. This enabled us to rapidly gain insights into this emerging pathogen population. We found that the PST population across the United Kingdom (UK) underwent a major shift in recent years. Population genetic structure analyses revealed four distinct lineages that correlated to the phenotypic groups determined through traditional pathology-based virulence assays. Furthermore, the genetic diversity between members of a single population cluster for all 2013 PST field samples was much higher than that displayed by historical UK isolates, revealing a more diverse population of PST.

**Conclusions:**

Our field pathogenomics approach uncovered a dramatic shift in the PST population in the UK, likely due to a recent introduction of a diverse set of exotic PST lineages. The methodology described herein accelerates genetic analysis of pathogen populations and circumvents the difficulties associated with obligate plant pathogens. In principle, this strategy can be widely applied to a variety of plant pathogens.

**Electronic supplementary material:**

The online version of this article (doi:10.1186/s13059-015-0590-8) contains supplementary material, which is available to authorized users.

## Background

Emerging and re-emerging diseases of humans, animals and plants pose a significant hazard to public health and food security. These threats can arise from newly discovered pathogens, such as the Middle East respiratory syndrome (MERS) coronavirus in humans [1], or novel host adaptation, as in zoonotic influenza [2]. Recent disease outbreaks in plants have been associated with expansions of pathogen geographic distribution and increased virulence of known pathogens, such as in the European outbreak of ash dieback [3] and wheat stem rust across Africa and the Middle East [4]. Independent of the host organism, the scale and frequency of emerging diseases have increased with the globalization and industrialization of food production systems [5]. Improved surveillance mechanisms and diagnostic tools are needed to rapidly respond to these emerging threats. With recent advances in DNA and RNA sequencing, bacteriologists and virologists are capitalizing on these technological advances by integrating high-resolution genotypic data into pathogen surveillance activities [6]. However, the application of genomics to emerging filamentous plant pathogens has lagged. Filamentous plant pathogens tend to have large genomes and are often obligate parasites that cannot be axenically cultured in the laboratory. The time-consuming and tedious protocols required to maintain these pathogens on their hosts have impeded the translation of genomic technologies into surveillance and diagnostics methods.

Traditional diagnostic tools for pathogens have been based on targeted cultures, PCR-based approaches and/or phenotypic evaluation of disease response in specific plant genotypes [7]. These methods detect only known pathogenic agents, can introduce bias, and can fail to recognize novel variants or races due to their narrow scope [8]. However, next-generation sequencing technologies can circumvent these limitations to provide a rich source of data for the development of surveillance and diagnostic tools. The high resolution of these approaches also enables exploration of the genetic determinants underpinning pathogenicity. Whole-genome sequencing has emerged as a preferred technology, especially for viruses with relatively small genomes (approximately 50 kb on average) [9], although this methodology is less tractable in pathogens with large genomes such as filamentous plant pathogens, which have genomes that range from 19 to 280 Mb [10]. Alternatively, RNA sequencing (RNA-seq), which focuses solely on the expressed fraction of the genome, reduces the sequence space of the sample and provides relevant transcriptome data for both the pathogen and host *in situ* [11].

Despite modern agricultural practices, diseases of the major food crops cause up to 15% pre-harvest yield loss [12]. Among these crops, wheat is a critical staple providing 20% of the calories and over 25% of the protein consumed by humans [13]. One of the major fungal diseases of wheat is yellow (stripe) rust caused by the obligate fungus *Puccinia striiformis* Westend. f. sp. *tritici* Eriks (PST) [14]. This disease is widespread across the major wheat-producing areas of the world and can cause significant reductions in both grain quality and yield in susceptible cultivars [15]. In the past decade, new PST races have emerged that are capable of adapting to warmer temperatures, have expanded virulence profiles, and are more aggressive than previously characterized races [16]. More recently, a series of PST races have arisen in Europe and overcome many of the major resistance genes in European germplasm [17]. For instance, in 2011 a race group collectively called ‘Warrior’ (based on the virulence of one of the initial variants of this group to the UK wheat variety Warrior) emerged as a serious threat to wheat production. However, the origin of this new race and its relationship with previously characterized races remain unclear.

An important first step towards the development of more effective surveillance and diagnostic tools is the availability of a draft reference genome and annotation. Cantu *et al*. [18] published a first draft sequence of PST isolate 130 (PST-130) with 22,185 annotated protein-coding sequences across the 64.8 Mb assembly. More recently, Zheng *et al*. [19] published a 110 Mb draft sequence of Chinese PST isolate CYR32 using a ‘fosmid-to-fosmid’ approach and annotated 25,288 protein-coding sequences. These genomic resources can be used to identify pathogenicity determinants, such as secreted effector proteins [20] that are recognized in certain host genotypes, where they induce an immune response that prevents disease progression. Avirulence effector proteins are under strong selective pressure to adapt in order to evade detection by the host plant immune system [21]. The signatures of adaptation and gene expression patterns of pathogen isolates with distinct virulence profiles can provide a powerful means of identifying specific avirulence/virulence proteins that can be used to track pathotypes at a national and international level. Furthermore, publication of these draft reference genomes also provides an opportunity to characterize pathogen populations at a considerably higher resolution and on a much wider scale through re-sequencing of PST isolates.

In this study, we developed a robust and rapid ‘field pathogenomics’ strategy, using transcriptome sequencing of PST-infected wheat leaves to gain insight into the population structure of an emerging pathogen. Our analysis uncovered a dramatic shift in the PST population in the UK and supports the hypothesis that recent introduction of a diverse set of exotic PST lineages may have displaced the previous PST populations. Our field pathogenomics approach circumvents the difficulties associated with less-tractable filamentous plant pathogens and can be applied to other emerging populations of pathogens.

## Results

### Genotyping pathogens and their hosts using RNA-seq of field-collected infected leaves

**Figure 1:**
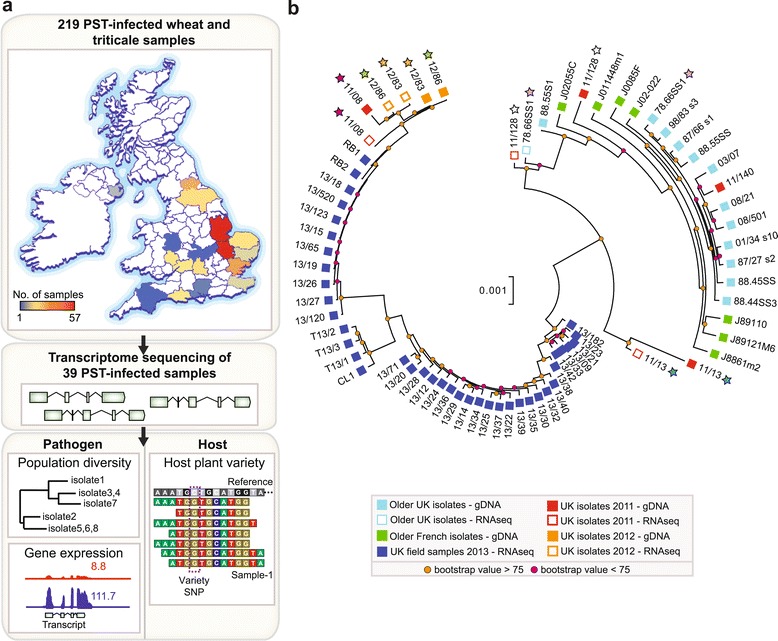
**PST field isolates belong to a diverse emergent lineage. (a)** A total of 219 samples of wheat and triticale infected with PST were collected from 17 different counties across the United Kingdom (UK) in the spring and summer of 2013. Transcriptome sequencing was carried out on 39 samples to generate transcript data from both the pathogen and host. For the pathogen, the data were used to assess the pathogen population diversity and differential gene expression. For the host, the data were used to confirm the host variety within a particular sample. SNP, single nucleotide polymorphism. **(b)** 2013 field isolates (dark blue squares) are distinct and highly diverse when compared with the older UK population (light blue squares). Phylogenetic analysis was undertaken using the third codon position of 5,610 PST-130 gene models (2,496,679 sites) with ≥80% breadth of coverage for all PST isolates using a maximum likelihood model. Stars indicate samples in which both the genome and transcriptome were sequenced from the same PST isolate.

To characterize the genotypic diversity of PST at the field level, we collected 219 samples of wheat and triticale infected with PST from 17 different counties across the UK in the spring and summer of 2013 (Figure 1a; Table S1 in Additional file 1). From these, we selected 35 PST-infected wheat samples from wheat varieties that spanned the resistance spectrum, and 4 PST-infected triticale samples (Table S1 in Additional file 1). Total RNA was extracted from each sample and subjected to RNA-seq analysis (Figure 1a). After filtering, an average of 37% (standard deviation 12.7%) reads aligned to the PST-130 reference genome [18], indicating that fungal transcripts account for a high percentage of the transcripts in PST-infected plant tissue (Table S2 in Additional file 1). To address whether each sample comprised a single PST genotype without considerable bias in allele-specific expression, we calculated the distribution of read counts for biallelic single nucleotide polymorphisms (SNPs), determined from alignment to the PST-130 genome. As a dikaryon, the PST mean of read counts at heterokaryotic positions is expected to have a single mode at 0.5, with two alternative alleles each representing one of the two haploid nuclei (Additional file 2) [22]. Based on the presence of only two alleles and the shape of the distribution being comparable to purified isolates when heterokaryotic SNPs were considered, we concluded that all samples likely represent a predominantly single genotype with little bias in allele expression (Additional files 3 and 4).

**Figure 2:**
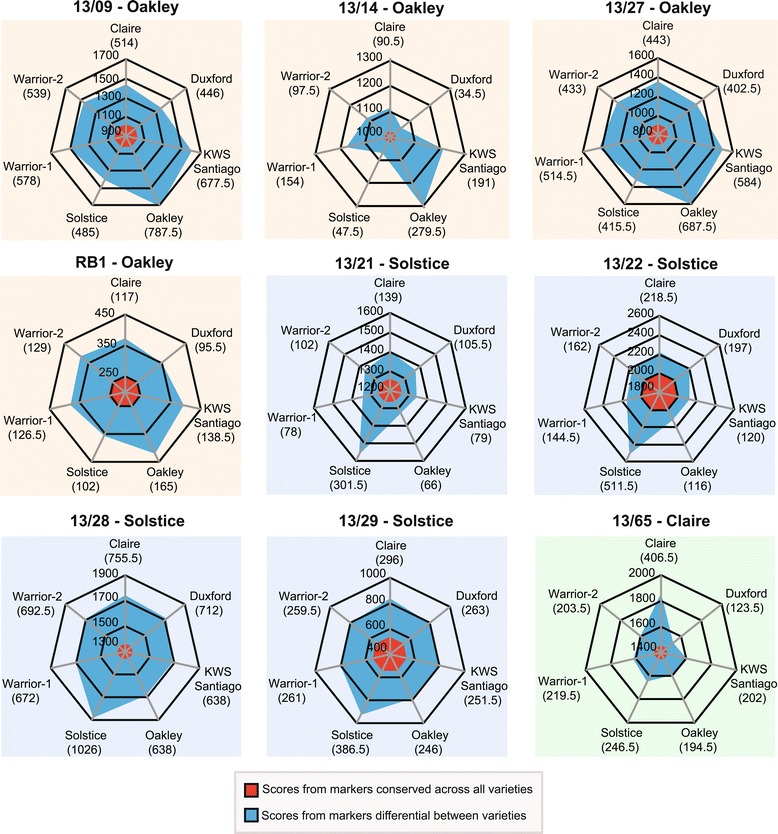
**Identification of wheat varieties using transcriptome data generated directly from PST-infected field samples.** A total of 18,162 SNP positions were used to differentiate wheat varieties. Each of the 18,162 SNP positions with ≥10× coverage was assessed for correlation against the available sequence data for seven wheat varieties. For each SNP position, if the PST-infected field sample matched the sequence at a SNP site for a particular variety (for example, variety = AA; field sample = AA) the position was scored 1, if the site only partially matched (for example, variety = AA; field sample = AC) then the position was scored 0.5, and if the site had no match (for example, variety = AA; field sample = CC) then the position was given a score of 0. For each sample, the total score was determined and visualized for each of the seven wheat varieties. Numbers in parentheses represent scores associated with differential markers for a particular wheat variety (blue shading). Monomorphic markers across all varieties are represented in red. Background colour and header relate to the reported variety for a given sample. Warrior-1, W1865; Warrior-2, W994.

We next used our data to confirm the wheat variety in a particular PST-infected sample. To this end, we extracted the wheat sequences flanking a set of 18,162 genetically mapped wheat SNPs (Table S3 in Additional file 1) [23]. Nine of the PST-infected wheat samples were collected from wheat varieties with identified SNPs (Donal O’Sullivan (University of Reading) and James Cockram (NIAB), personal communication) and for each of these samples, reads were independently aligned against the wheat sequences extracted above. Each of the 18,162 SNP positions with ≥10× coverage was then assessed for correlation against the available sequence data for seven wheat varieties. This analysis confirmed the wheat variety recorded at the point of sample collection as the most likely variety for all nine PST-infected wheat samples (Figure 2). Furthermore, for samples taken from the wheat variety Oakley, the second highest matching variety was KWS Santiago, whose parents are Sherbourne and Oakley. Oakley has been used widely in the parentage of various wheat varieties as reflected by the level of similarity between PST-infected Oakley samples and all other varieties (Figure 2). This analysis demonstrates that the transcriptomic data from PST-infected field samples can be used successfully to determine the host wheat variety.

### A dramatic shift in the PST population in the UK

To determine the relationship between the 2013 PST field isolates and previously prevalent PST populations, the genomes of 14 UK and 7 French purified PST isolates collected between 1978 and 2011 were sequenced using an Illumina whole-genome shotgun approach (Table S4 in Additional file 1). After filtering, reads were independently aligned to the PST-130 reference genome. Phylogenetic analysis was undertaken using the third codon position of 5,610 PST-130 gene models (2,496,679 sites) with ≥80% breadth of coverage for all PST isolates using a maximum likelihood model. This analysis illustrated that 13 of the 14 historical UK PST isolates and all French isolates clustered together in a single clade with little genetic variation (Figure 1b). By contrast, the PST field isolates collected in 2013 were distantly related to the older UK population, and included several diverse lineages. Furthermore, a subset of 11 of the 39 PST 2013 field isolates were also genetically similar to a characterized ‘Warrior’ type PST isolate from 2011 (PST-11/08; Figure 1b). This indicates that a diverse PST population that contained the ‘Warrior’ pathotype was prevalent across the UK in 2013.

With the first record of the ‘Warrior’ pathotype occurring in the UK in 2011, we decided to investigate the distribution of this lineage further by sequencing the genome of two purified PST isolates with known virulence profiles from 2011 and two from 2012 [24]. After filtering, reads were aligned to the PST-130 reference genome. Phylogenetic analysis revealed that two PST isolates from 2011 (PST-11/128 and PST-11/13) were more closely related to the older UK population, whereas the remaining 2012 isolates clustered within the ‘Warrior’ type lineage (Figure 1b). To further support the topology of the phylogenetic tree, we extracted RNA from a susceptible wheat variety infected independently with six PST isolates (PST-78/66, PST-12/86, PST-12/83, PST-11/13, PST-11/128 and PST-11/08) that were also subjected to genome sequencing. The distribution of biallelic SNPs, from alignment to the PST-130 genome, confirmed that each sample comprised predominantly a single PST genotype without considerable bias in allele-specific expression (Additional file 5). When SNP sites with sufficient depth of coverage in both the genomic and RNA-seq samples were compared, an average of 99.78% were identical between the genomic and RNA-seq datasets (Table S5 in Additional file 1). This indicates that allele-specific gene expression had a negligible effect on the topology of the phylogenetic tree. This analysis further supports the recent emergence of a diverse PST population that may have now displaced the previous PST population in the UK.

### A genetically diverse PST population in the UK in 2013

**Figure 3:**
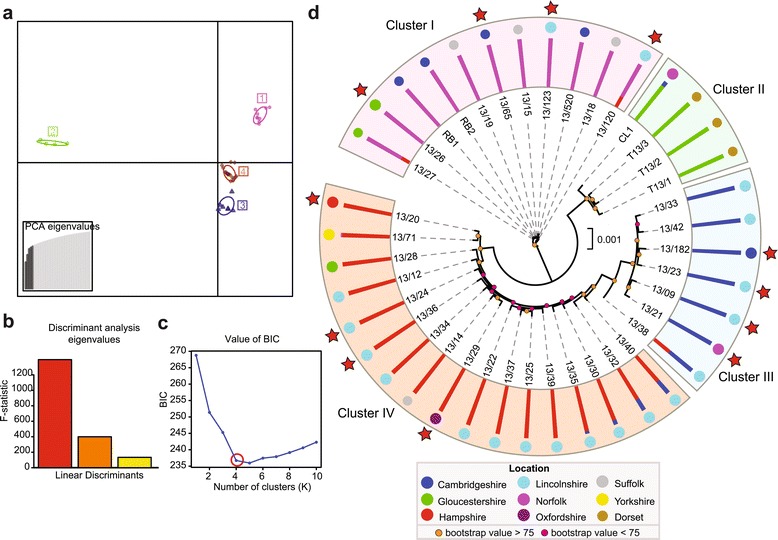
**The 2013 PST field isolates are highly diverse and group genetically into four distinct population clusters. (a)** Scatterplot using the first two principal components (Y-axis and X-axis, respectively) of the discriminant analysis of principal components (DAPC) analysis of 34,806 synonymous single nucleotide polymorphism (SNP) sites. Each symbol represents a single PST isolate, coloured according to assignment to one of four population clusters. All four population clusters are clearly separated by DAPC analysis. **(b)** The first three eigenvalue components from the DAPC analysis, supporting the maintenance of three discriminant functions in the DAPC analysis. **(c)** The optimal predicted number of population clusters K for the dataset is four. The Y-axis corresponds to the Bayesian information criterion (BIC), a goodness-of-fit measurement calculated for each K. The elbow in the BIC values (K = 4) indicates the optimal number of populations. **(d)** Phylogenetic analysis using a maximum likelihood model (2,513,246 sites) and Bayesian-based clustering of 34,806 synonymous SNP sites classified PST field isolates into four population clusters. All PST isolates sampled from triticale clustered within a single genetically distinct lineage, cluster II. Bar charts represent STRUCTURE analysis, with each bar representing estimated membership fractions for each individual. Stars highlight isolates purified for virulence profiling. Coloured circles represent UK counties in which samples were collected (Table S1 in Additional file 1).

To elucidate the population structure among the 39 PST 2013 UK field isolates, we generated a list of 34,806 synonymous SNP sites, of which 34,764 were biallelic. We used multivariate discriminant analysis of principal components (DAPC) with the 34,764 biallelic SNP sites to define the population structure and identify groups of genetically related PST isolates. The Bayesian information criterion supported the division of PST isolates into four population clusters, which were clearly distinct in a scatterplot of the five principal components of the DAPC (Figure 3a,b). In addition, Bayesian-based clustering of the full set of 34,806 synonymous SNP sites using the program STRUCTURE classified the PST isolates into four population clusters (Figure 3c,d) that differed only in the partitioning of two isolates (PST-13/120 and PST-13/27) compared with the DAPC assignment (Additional file 6). Phylogenetic analyses were also undertaken using the third codon position of 5,713 genes (2,513,246 sites) with ≥80% breadth of coverage for all PST isolates using a maximum likelihood model. This analysis supported the assignment of PST isolates to the four population clusters as reported by the Bayesian-based clustering method (Figure 3d). Cluster-specific SNPs were converted into PCR-based assays and shown to differentiate the PST lineages (Table S6 in Additional file 1; Additional file 7). Furthermore, within the PST field samples collected in 2013, all PST isolates sampled from triticale clustered within a single genetically distinct lineage (Figure 3d, cluster II), potentially indicating a degree of host specificity within the PST population in the UK.

**Figure 4:**
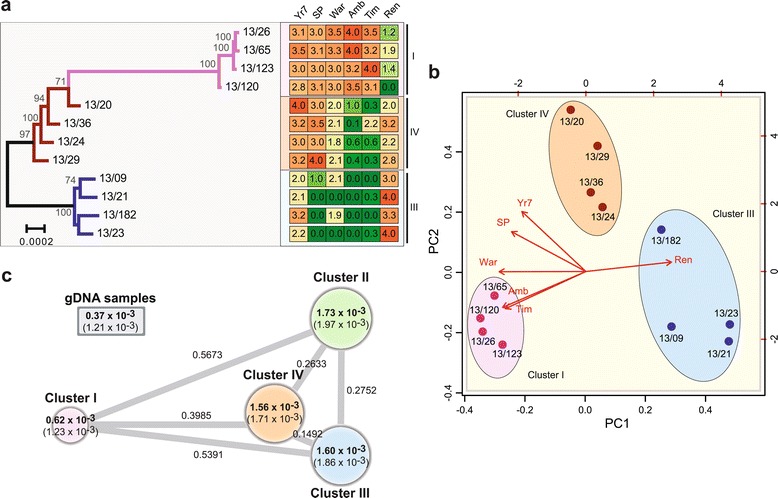
**The 2013 PST field isolates identified on wheat show correlation between genetic and phenotypic profiles. (a)** The population substructure illustrated by phylogenetic analysis of 12 PST isolates using a maximum likelihood model (6,479 genes; 2,792,462 sites) correlated with the virulence profiles of the isolates. Virulence profiles for each population cluster were largely conserved between members of the same population cluster, but differed from those of members of other population clusters. Four PST isolates from each of the three population clusters derived from PST-infected wheat samples were inoculated on a series of 39 different wheat varieties and the six discriminative results are shown: *Yr7*, AvocetS-*Yr7* near isogenic line; Sp, Spaldings Prolific; War, Warrior; Amb, Ambition; Tim, Timber; Ren, Rendezvous. Disease severity was recorded 16 to 20 days post-inoculation: 0 (green) to 4 (red) scale with 0 to 2 resistant and 2 to 4 susceptible. Roman numerals represent population clusters. **(b)** Principal component analysis of the virulence profiles of the 12 PST isolates supports the separation of isolates into three distinct groups based on their phenotypes, which correlates with their genetic partitioning. **(c)** The degree of genetic diversity between members of a single population cluster for all 2013 PST field samples was much greater than that displayed by older UK isolates collected between 1978 and 2011. Circle size represents the degree of nucleotide diversity enclosed; standard deviation is given in parentheses. Branch lengths represent *F*_*ST*_ values stated alongside.

Next, to determine if the observed population structure was reflected in the phenotypic characteristics of the PST 2013 field isolates, we purified and cultured a subset of isolates for virulence profiling. Four PST isolates from each of the three population clusters derived from PST-infected wheat samples were inoculated on a series of 39 differential wheat varieties. Disease severity was recorded 16 to 20 days post-inoculation (Table S7 in Additional file 1). Phylogenetic analysis of the 12 PST isolates using a maximum likelihood model (6,479 genes; 2,792,462 sites) was combined with their virulence profiles to assess correlations between the population substructure and pathology data. This analysis revealed distinct phenotypic characteristics for each population cluster that were different from members of other population clusters, but were largely conserved between isolates of a similar genetic background (Figure 4a). Furthermore, principal component analysis of the phenotypic characteristics supported the clear division of the isolates into three phenotypic groups that correlated directly with the genetic population clusters (Figure 4b). This reflects a clear association between the genotypic and phenotypic diversity displayed by the 2013 PST field isolates.

Cluster I isolates displayed the least phenotypic diversity between PST isolates. This correlated with much lower nucleotide diversity between members of this cluster compared with other clusters (Figure 4c). Overall, however, the degree of genetic diversity between members of a single population cluster for all 2013 PST field samples was much higher than that displayed by the older UK and French isolates collected between 1978 and 2011, excluding PST-11/08 (Figure 4c). Substantial genetic differentiation was also identified in all pair-wise comparisons of the four population clusters, with *F*_*ST*_ values ranging from 0.1492 to 0.5673 (Figure 4c). The variation in gene expression between members of a population cluster did not influence the calculation of genetic diversity (Additional files 7 and 8). Taken together, this supports the hypothesis that the new UK PST population is derived from a highly diverse founder population.

### Polymorphic and differentially expressed effector candidates can be linked to the virulence profiles of the PST 2013 field isolates

**Figure 5:**
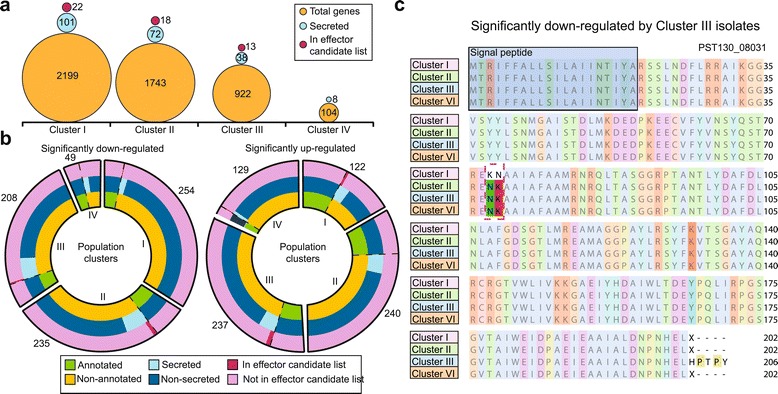
**Polymorphic and differentially expressed effector candidates can be linked to the virulence profiles of the PST 2013 field isolates. (a)** Polymorphism analysis highlights cluster-specific effector candidates. Circles represent the number of genes with non-synonymous mutations in each category. **(b)** Differential expression analysis identifies cluster-specific differentially expressed effector candidates. Roman numerals represent population clusters. **(c)** PST130_08031 is a previously identified effector candidate that was significantly down-regulated by isolates in cluster III and had two amino acid substitutions that were specific and conserved among cluster I isolates. The five carboxy-terminal amino acids were not defined due to poor coverage.

We also used our field pathogenomics approach to look for two signatures of adaptation, namely mutation and differential gene expression, by treating all isolates within a population cluster as replicates in the analysis. Specifically, we sought to identify potential effector proteins and link these to the distinct virulence profiles within the 2013 PST population. First, to identify polymorphic effector candidates, we discriminated 21,217 homokaryotic and heterokaryotic SNP sites that induced non-synonymous substitutions from alignment of all 39 PST 2013 field isolates against the PST-130 reference genome. Of these non-synonymous sites, we identified 10,158 SNP sites where the amino acid residue was conserved among all members of a single population cluster with coverage in the region, but differed from the amino acid encoded by all members from at least one other population cluster (Table S8 in Additional file 1). These 10,158 SNP sites were dispersed among 4,633 genes that displayed cluster-specific unique amino acid substitutions, of which 177 had detectable secretion signals (Figure 5a). Using the most highly ranked PST effector candidates from our previous study [20], we identified 42 genes that encoded cluster-specific polymorphic proteins that displayed features typical of characterized effector proteins (Figure 5a).

Next, to assess whether the gene expression profiles of the 39 PST field isolates could be associated with cluster-specific disparity in virulence profiles, reads from each isolate were aligned independently to the PST-130 genome. Differential expression analysis was conducted after normalization to identify genes that were significantly differentially regulated between the four population clusters (false discovery rate <0.05; *P*-value <0.05). All isolates within each population cluster were used as replicates in the analysis (Table S9 in Additional file 1; Additional file 9). Of the genes that were identified as significantly down- and up-regulated for all isolates within a particular population cluster, between 8.5 and 45.9% could be annotated with potential structural or enzymatic functions (Figure 5b; Additional file 9). Of those that were not annotated, an average of 16.7% (standard deviation 9.6%) were predicted to encode proteins with detectable secretion signals (Figure 5b). Furthermore, we identified 10 up-regulated and 9 down-regulated genes that were among the most highly ranked PST effector candidates from our previous study (Figure 5b). One of these candidates, PST130_08031, was significantly down-regulated by isolates in cluster III and had two amino acid substitutions that were specific and conserved among cluster I isolates (Figure 5c; Additional file 9).

## Discussion

### Exploiting transcriptome sequencing for surveillance and population analysis of (re)-emerging pathogens

Human, animal and plant pathogens necessitate constant monitoring to preserve public health and food security. With the advent of next-generation sequencing technologies, it is now possible to integrate high-resolution DNA and RNA sequencing into pathogen surveillance programs. However, many pathogens cannot be axenically cultured, limiting access to pure DNA and RNA preparations. Furthermore, large-scale population analysis of fungal pathogens by whole-genome sequencing remains limited by the lengthy processes associated with purification and multiplication of isolates for high molecular weight DNA extraction and the cost of sequencing large genomes. We have developed an approach for pathogen population surveillance based on high-resolution transcriptome data acquired directly from field samples of pathogen-infected wheat and triticale. Even though the analyzed samples consist of a mixture of pathogen and host RNA, we recovered enough pathogen sequences for analysis. Also, the RNA-seq data were deep enough for reliable genotypic characterization. Similar approaches using shotgun genome sequencing could have been problematic due to the large size of the genome of wheat (approximately 17 GB) compared with that of PST (approximately 110 Mb) [19,25]. Our approach also captures the PST population directly from the field and negates any biases that might be caused by purification and multiplication of the pathogen in the laboratory, a lengthy process that can impose artificial selection on the pathogen.

Using field pathogenomics, we could detect only a single PST genotype within each lesion. Furthermore, using comparative analysis of RNA-seq and genomic sequence data from six independent PST isolates (PST-78/66, PST-12/86, PST-12/83, PST-11/13, PST-11/128 and PST-11/08), we were able to confirm that allelic-specific expression between the two PST nuclei had minimal effect on genotypic analysis. Together these results demonstrate that RNA-seq analysis of PST-infected plant material is a useful approach for accurately genotyping isolates of PST directly from the field. However, our findings contrast with studies of *Mycosphaerella graminicola* on wheat and *Rhynchosporium secalis* on barley, where co-infection with multiple genotypes is common [26,27]. Analyses of field pathogenomics data may be more complex in such pathosystems.

Whilst effectively capturing pathogen diversity, transcriptome sequencing of infected host tissue can also be leveraged to assess the genotype of the host. The availability of high-throughput SNP chips for wheat [23] and SNP marker information for the majority of wheat varieties in the UK [28] (and elsewhere) provides an unprecedented opportunity to exploit sequence data to confirm outbreaks on particular wheat varieties and look for associations between pathogen genotypes and host pedigrees. In this study, we developed an accurate system to associate samples from known wheat varieties with their corresponding SNP markers. In the future, this will provide a rapid means of confirming whether previously resistant wheat varieties have indeed been broken by virulent races of the pathogen, using samples submitted directly to national pathology surveys. This would reduce delays associated with current protocols, which include pathogen propagation, subsequent virulence profiling and confirmation of a specific wheat variety using protein gels from harvested grains or similar distinctness, uniformity and stability assessments [29].

### Integration of high-resolution genotyping into traditional pathogen surveillance surveys

Traditionally, the surveillance of rust fungal pathogens in agroecosystems has hinged on field biology and race pathotype surveys to provide phenotypic information on pathogen diversity [30]. However, assessments of genotypic diversity are not included routinely and when employed are restricted to just a handful of markers such as simple sequence repeats or amplified fragment length polymorphisms [31]. Our field pathogenomics approach enables the integration of high-resolution genotypic data into pathogen surveillance activities. For instance, more than 2 million nucleotide positions were used to assess PST population diversity in this study. These high-resolution genotypic data are vital to improve our understanding of the genetic substructure within a population, which provides essential information on the evolutionary forces that drive pathogen evolution within an agroecosystem. This study uncovered four genetically distinct lineages within the UK PST population, and each of these lineages had unique virulence profiles revealing a direct link between genotype and pathotype. Although such a correlation has been reported for rust fungi [32,33], our findings contrast to distantly related filamentous plant pathogens such as *Magnaporthe oryzae* [34] and *Colletotrichum lindemuthianum* [35] where a relationship between genotype and pathotype has not been detected.

The time-consuming nature of traditional surveillance methods limits the number of PST isolates assessed each year. For instance, in the UK, a target number of 25 PST-infected wheat samples are tested each year, specifically focusing on wheat varieties with a previous record of good resistance in the field. With new PST pathotypes/genotypes arising on susceptible varieties by mutation, recombination or through exotic incursions, it is unlikely that a new pathotype would be detected in a timely fashion by the current surveillance system. Furthermore, an exotic isolate that displays similar phenotypic characteristics to a subset of the existing population would not be recognized as such. In this study, we uncovered a group of PST isolates (population cluster III) that displayed identical phenotypic characteristics to a subset of the old UK population, but in fact belonged to a new emergent lineage that appears to be new to the UK. None of these isolates would have been identified as belonging to an emergent lineage based on phenotypic data alone. However, such population shifts may bear significance on disease incidence as the new population may carry important epidemiological traits other than pathogen virulence. Rapid and systematic application of field pathogenomics should transform current disease surveillance systems by generating high-resolution genotypic information (Additional file 10) that inform disease incidence models, agronomic practices, and the selection of PST isolates for subsequent labor-intensive phenotypic characterization.

### Using effector-specific markers to track pathotype dispersal

The emergent PST population in the UK is now dominated by a number of newly selected, virulent clones that are adapted to an array of widely cultivated wheat varieties. By revealing genotype/pathotype-specific polymorphisms, the data we generated could prove useful in identifying candidate avirulence effectors that contribute to a pathogen’s ability to evade recognition on particular host genotypes. Herein, our analysis identified a small number of candidate effector genes with conserved mutations or expression profiles between members of the same population cluster that shared similar virulence profiles. Ultimately, such information could be used to develop polymorphic markers to track the long-distance migration of pathotypes across wheat growing regions.

### Field pathogenomics reveals a shift in the PST population in the UK

We uncovered a dramatic shift in the PST population that could have serious implications for wheat production in the UK. Whilst there have been widespread reports of recent changes in the PST population based on phenotypic characteristics [17], we report a comprehensive genetic analysis of this emergent PST population. Plant-pathogenic fungi rely predominantly on recombination and mutation as the evolutionary forces that drive the emergence of new races and pathotypes [36]. However, within a pathogen population, gene and genotype flow can shape the population substructure as propagules are exchanged between geographically separated epidemiological areas [36]. Given the clonal population structure of PST in northwestern Europe, mutation and genotype flow are the primary inducers of diversity [32]. The fact that none of the 2013 PST field isolates showed genetic similarity to the great majority of the older UK population (collected between 1978 and 2011; excluding PST-11/08) indicates that the 2013 population is likely an exotic PST population that appears to have displaced the previous population. Furthermore, the highest level of genetic diversity between the four emergent PST lineages (*F*_*ST*_ ranging from 0.1492 to 0.5673) was similar to that detected using simple sequence repeat markers and comparing PST isolates from different continents [37]. This is indicative of distant ancestry or relatively low levels of gene flow between these emergent UK PST lineages. Based on this evidence, we hypothesize that the change in PST population structure may have arisen from exotic incursions from multiple sources over recent years. Future studies will focus on defining the origin(s) of this PST population.

A subset of the emergent PST population we characterized displays the ‘Warrior’ pathotype that was first detected in 2011 in the UK and is virulent on an array of previously resistant wheat varieties, including Alchemy, Warrior, and Claire [24]. Our findings illustrate how pathogen genotype flow can trigger abrupt changes in the landscape of wheat genetic resistance to yellow rust. Breeders are now at a crossroads in the UK, with few sources of yellow rust disease resistance available and the prospect of new varieties being rapidly taken off the official recommended list due to poor yellow rust resistance, as happened with Torch (1 year on the recommended list) and Warrior (3 years). With anthropogenic activities having a marked influence on the size of genetic neighborhoods [38], pathogen genotype flow is no longer dependent on life history traits and natural dispersal alone. The next step will be to define the boundaries of these ever-expanding genetic neighborhoods to inform surveillance strategies and breeding programs that need to take into account the full pathogen population within an isolated genetic neighborhood to breed for durable resistance.

### Exploring the origin of PST diversity in the UK

The 2013 PST isolates displayed a much higher degree of nucleotide diversity when compared with the older UK population. This reflects an increase in PST evolutionary potential in the UK pathogen population that could enhance their ability to overcome genetic resistance in the host. Given that the highest levels of PST genotypic diversity have been reported in the Himalayas and neighboring regions, it is possible that the emerging PST population is derived from one or more migration events from a geographic area with high sexual reproduction rates and a recombinant population structure [37]. This is further supported by similarity in pathotypes between one lineage (cluster I) of the emergent UK population and those previously reported for exotic PST isolates [39]. For instance, three Chinese isolates that were collected in 2004 and a Nepalese isolate from 2008 were shown to be virulent on the wheat variety Spaldings Prolific [40], which is a key determinant for the cluster I (‘Warrior’) pathotype [17]. Furthermore, Ali *et al*. [40] previously classified two Chinese isolates collected in 2001 as belonging to the Northern French genotypic group (G1). Future studies will focus on comparative sequence analysis between the PST isolates reported herein and global isolates of PST to determine the specific geographic origin(s) for this diverse PST population in the UK.

### The future of genomics-enabled plant pathogen surveillance systems

The agronomic consequences of long-distance pathogen migration are currently unpredictable. Although a pathogen population may not pose a significant threat to crop production in the country of origin, it can have devastating consequences in a new environment. For instance, in 2013 a severe stem rust epidemic in Ethiopia was caused by a race similar to those detected in Egypt, Germany and Turkey between 2007 and 2013. However, despite the widespread devastation reported in Ethiopia, other countries reported no negative effect of this race on wheat production. This episode illustrates the importance of global pathogen surveillance networks, to enable early warning systems that assess the threat of pathotypes to all crop genotypes planted within a single genetic neighborhood. Field pathogenomics provides the means to generate enough markers to comprehensively genotype the PST population. High-resolution SNP marker arrays would allow tracking pathogen dispersal on a global scale and clear definition of the pathogen population genetic structure. The approach reported herein uses attenuated PST-infected field samples, thereby negating the limitations associated with movement of live samples. Whilst genotyping is undertaken in state-of-the-art molecular laboratories, the complementary virulence profiling can be carried out in national centers, thereby preventing any threat posed by transportation of live samples between countries. Once genotypic information is generated, subsequent phenotypic characterization can focus on the most notable and representative samples ensuring the best possible use of limited national resources.

## Conclusions

In this study, we developed a robust and rapid method based on RNA sequencing directly from infected host samples to gain insight into emerging pathogen populations. Field pathogenomics should be applicable to surveillance of many pathogens besides wheat rust pathogens, and could contribute to addressing human, animal, and plant health issues. Our approach enabled us to discover a dramatic shift in the UK PST population in 2013 essentially months after collecting the field samples. The emergent PST population has high levels of genetic diversity compared with historical UK isolates and appeared to be unrelated to the older population. This led us to conclude that the 2013 PST population was most probably derived from the recent introduction into the UK of diverse assemblage of exotic PST lineages, and that these introduced lineages may have rapidly displaced the previous PST population. Such detailed knowledge of population shifts and dynamics is important for our understanding of emerging plant diseases and has consequences for the management of such diseases.

## Materials and methods

### Whole-genome and transcriptome sequencing of PST-infected wheat and triticale

A total of 219 single lesion leaf samples of PST-infected wheat and triticale were collected directly from the field and stored in RNA later solution at 4°C (Life Technologies, Paisley, UK). The single lesion consisted of a 2 to 3 cm leaf section taken from a single infection site. Total RNA was extracted from 39 of these samples using the Qiagen RNeasy Mini kit according to the manufacturer’s instructions (Qiagen, Manchester, UK). In addition, we extracted RNA in a similar manner from infected leaves of susceptible wheat variety Vuka inoculated independently with six PST isolates (PST-78/66, PST-12/86, PST-12/83, PST-11/13, PST-11/128 and PST-11/08). The quantity and quality of RNA extracted were assessed using the Agilent 2100 Bioanalyzer (Agilent Technologies, Edinburgh, UK). cDNA libraries were prepared using the Illumina TruSeq RNA Sample preparation Kit (Illumina, Cambridge, UK). Library quality was confirmed before sequencing using the Agilent 2100 Bioanalyzer (Agilent Technologies, Edinburgh, UK). Libraries were sequenced on the Illumina GAIIx at The Sainsbury Laboratory (for RB1 and RB2) or the Illumina HiSeq machine at The Genome Analysis Centre, UK. Adapter and barcode trimming and quality filtering were carried out using the FASTX-Toolkit. The 76-bp (GAIIx) or 101-bp (HiSeq) paired-end reads were aligned to the PST-130 assembly [18] using the TopHat package (version 1.3.2) and Bowtie alignment program (version 0.12.7) with default parameters [41,42]. A similar approach was used for whole genome sequencing of PST isolates, except that gDNA was extracted for each isolate from dried urediniospores using the CTAB method as described by Chen *et al*. [43] and DNA quantity was confirmed using the Qubit 2.0 Fluorometer. DNA libraries were prepared using the Illumina TruSeq DNA Sample preparation Kit (Illumina, Cambridge, UK). Sequencing of all gDNA samples was carried out on an Illumina HiSeq machine at The Genome Analysis Centre, UK, generating 101-bp paired-end reads which were aligned to the PST-130 assembly [18] using BWA with default parameters [44]. The Illumina reads from all RNA-seq and gDNA runs were deposited in the short read archive (GenBank; PRJNA256347 and PRJNA257181).

### Identifying the wheat variety in PST-infected field samples

First, from a set of 90,000 high-density wheat SNPs, 18,162 genetically mapped wheat SNPs were extracted [23]. Up to 100 bp up- and down-stream of each SNP site were extracted from the wheat chromosome arm survey sequence [45] to create a reference for subsequent sequence alignments. Nine PST-infected field samples were collected on wheat varieties with known varietal SNP information (Donal O’Sullivan (University of Reading) and James Cockram (NIAB), personal communication). Reads from each of these nine samples were independently aligned to the wheat genome sequences extracted above using the TopHat package (version 1.3.2) and Bowtie alignment program (version 0.12.7) with default parameters [41,42]. Each of the 18,162 SNP positions with ≥10× coverage was then assessed for correlation against the available sequence data for the seven wheat varieties. For each SNP position, if the PST-infected field sample matched the sequence at a SNP site for a particular variety (for example, variety = AA; field sample = AA) the position was scored 1, if the site only partially matched (for example, variety = AA; field sample = AC) then the position was scored 0.5, and if the site had no match (for example, variety = AA; field sample = CC) then the position was given a score of 0. For each sample, the total score was determined and visualized for each of the seven wheat varieties.

### Calling single nucleotide polymorphisms

BAM files were sorted and indexed, and SNPs determined using raw allele counts for each position that were obtained using pileup from SAMtools [46]. Heterokaryotic sites were identified as sites with allelic frequencies ranging from 0.2 to 0.8. Homokaryotic sites were those with allelic frequencies below 0.2 or above 0.8. For both hetero- and homokaryotic sites to be reported, they had to satisfy a minimum depth of coverage of 20× for RNA-seq data and 10× for genomic DNA data. Read frequencies were calculated for biallelic heterokaryotic SNP sites and plotted using ggplot2 in R [47]. Homokaryotic and heterokaryotic SNP sites that induced synonymous and non-synonymous substitutions were identified using SnpEff, version 3.6 [48].

### Phylogenetic analysis of the historical and current UK PST population

All phylogenetic analysis of PST isolates was conducted using a maximum likelihood approach. First, for both genomic and RNA-seq samples, nucleotide residues that differed from the PST-130 reference were identified and recorded if they satisfied a minimum of 10× or 20× depth of coverage, respectively. Next, sites that were identical to the reference were recorded when they satisfied a minimum of 2× depth of coverage. Finally, these sites were used to generate synthetic gene sets for each isolate and genes with a minimum of 80% breadth of coverage for all samples in a comparison were selected. The third codon position of these genes was then used to build maximum likelihood trees using RaxML 7.0.4 with 100 replicates using the rapid bootstrap algorithm [49]. Phylogenetic trees were visualized in MEGA6.06 [50]. For the RNA-seq samples, results from STRUCTURE analysis were incorporated into the phylogenetic tree using iTOL [51].

### Population structure analysis of PST field isolates in the UK in 2013

Genetic differentiation of the 39 PST field isolates was examined using the Bayesian model-based approach implemented in the software STRUCTURE, version 2.3.4 [52] via the python StrAuto program, version 3.1 [53]. First, a list of 34,806 sites that introduced a synonymous change in at least one isolate was generated. Then, the nucleotide at this position was extracted for all 39 RNA-seq samples. The ‘admixture’ model was used with three replicates of 200,000 Markov Chain Monte Carlo generations for K = 1 to 10, where K is the number of populations. For each run the first 100,000 generations were discarded as burn-in before collecting data. To identify the K value the average log probability (LnP(D)) of each K value was calculated [52].

The genetic differentiation of the 39 field isolates was further assessed using the multivariate DAPC within the adegenet package [54]. First, 34,764 biallelic SNP sites that introduced a synonymous change in at least one isolate were identified. Using these data, principal component analysis was carried out to summarize genetic variation between and within potential population clusters. The optimum number of clusters was determined as the one showing the lowest Bayesian information criterion. DAPC analysis was then used to assign individuals to each of the population clusters.

### Assessing diversity within and between PST population clusters

To assess the genetic diversity both within and between PST population clusters, all heterokaryotic and homokaryotic SNPs determined above from individual alignment of each isolate to the PST-130 reference were incorporated into a synthetic gene set for that isolate. The synthetic genes were combined for all PST field isolates within a population group, and genes with >80% breadth of coverage for all isolates were selected. To calculate the degree of nucleotide diversity between isolates of a single population group, the degree of polymorphism between these gene sets was calculated using the DnaSP software package, version 5.10.1 [55]. To determine the proportion of total genetic variance attributable to inter-population differences, the 34,806 sites that introduced a synonymous change in at least one isolate were used as input in the program Genepop version 4.2 [56] to calculate the Wright’s *F*_*ST*_ statistic.

### Virulence profiling of PST isolates

Virulence phenotyping of PST isolates was based on the reactions of wheat cultivars possessing known resistances to PST, together with a number of cultivars possessing resistances which have not yet been fully described. Tests were carried out on seedlings under controlled environment conditions [57], with infection types being assessed on the first seedling leaf using a 0 to 4 scale. Infection types 3 and 4 were considered to represent a compatible interaction between host genotype and pathogen isolate, indicating the absence of *Avr* alleles (that is, virulence) at the corresponding locus in the pathogen. The host resistance genes covered by the differential set were *Yr1*, *Yr2*, *Yr3*, *Yr4*, *Yr5*, *Yr6*, *Yr7*, *Yr8*, *Yr9*, *Yr10*, *Yr15*, *Yr17*, *Yr24*, *Yr25*, *Yr32* and the resistance in Spaldings Prolific. Other discriminating differentials included the cultivars Robigus, Solstice, Timber, Warrior, Ambition, and Rendezvous. To distinguish the internal structure and variance within the pathology data, the scores associated with the reactions of each isolate on the differential wheat cultivars were used for principal component analysis in R [58].

### Gene expression analysis between PST population clusters

Quantification of reads mapping to the PST-130 gene set from the 39 PST field isolates was determined using the program HTSeq-count [59]. Next, the Fisher’s exact test, implemented as part of the edgeR package [60], was used to identify genes that were significantly differentially regulated between the four population clusters (false discovery rate <0.05; *P*-value <0.05). All isolates within each population cluster were used as replicates in the analysis to (1) limit the influence of environmental factors on the expression profiles, as samples were collected at various sites throughout the season, and (2) to link gene expression profiles to the virulence profiles that were unique to these genotypic groups. To identify potential effector proteins with signatures of adaptation such as mutation and variation in gene expression profiles, we focused on accessing those that were ranked the highest in our previous effector mining study [20]. Previously, we clustered protein sequences based on sequence similarity and ordered the resulting protein families based on the association of known effector features and PST-specific annotation [20]. This resulted in overall scores for each family that reflected their likelihood of containing potential effector proteins [20]. Those within the top 100 protein families were considered herein.

### KASP assays

Primers were designed with primer3 version 2.3.5 [61] carrying standard FAM or HEX compatible tails (FAM tail: 5′ GAAGGTGACCAAGTTCATGCT 3′; HEX tail: 5′ GAAGGTCGGAGTCAACGGATT 3′) and with the target SNP at the 3′ end. Oligonucleotides were ordered from Sigma-Aldrich (Gillingham, UK) and primer mixes were as recommended by the manufacturer (46 μl dH_2_O, 30 μl common primer (100 μM), and 12 μl each tailed primer (100 μM); LGC Genomics, Teddington, UK). Assays were carried out as described previously [62] with the following modifications: 4 μl reactions were used (composed of 2 μl template (10 to 20 ng DNA), 1.944 μl V4 2× Kaspar mix, and 0.056 μl primer mix)), PCR cycling was performed in an Eppendorf Mastercycler pro 384 and 384-well optically clear plates (catalogue number E10423000, Starlab, Milton Keynes, UK) were read on a Tecan Safire plate reader. Data analysis was performed manually using Klustercaller software (version 2.22.0.5, LGC).

## Additional files

Additional file 1:
**Contains supplementary Tables S1 to S9.** Microsoft Excel Workbook containing nine worksheets. Table S1: all PST-infected field samples collected in 2013. Table S2: fungal transcripts account for a high percentage of the transcripts in PST-infected plant tissue. Table S3: wheat variety SNPs from iSelect wheat SNP chip (Wang *et al*. [23]). Table S4: virulence profiles of PST isolates subjected to full genome sequencing. Table S5: comparison of SNP sites between genomic and RNA-seq datasets generated from the same PST isolate. Table S6: genotype data for nine PST field samples generated from KASP assays. Table S7: full seedling virulence tests of selected PST isolates. Table S8: non-synonymous SNP sites where the amino acid residue was conserved among all members of a single population cluster with coverage in the region, but differed from the amino acid encoded by all members of at least one other population cluster. Table S9: differential expression analysis based on Fisher’s exact test of all pair-wise comparisons of population clusters. Additional file 2:
**Distribution of biallelic read counts.** **(a,c)** Read frequency at biallelic single nucleotide polymorphisms (SNPs) for a gDNA sample known to consist of multiple mixed genotypes. The presence of several alleles with three copies and the presence of an uneven frequency distribution are indicators of more than a single genotype in the sample. **(b,d)** Read frequency at biallelic SNPs for a purified gDNA sample that consists of only a single PST genotype. The even frequency distribution and presence of only two alleles support the presence of a single genotype in the sample. The mode for each distribution is given in parentheses. Additional file 3:
**Distribution of biallelic read counts for purified UK isolates subjected to full genome sequencing.** The 16 purified UK PST isolates that were selected for genome sequencing show varying patterns of read frequencies for biallelic single nucleotide polymorphisms. The mode for each distribution is given in parentheses. Additional file 4:
**All PST-infected plant samples consist of a single PST genotype.** Distribution of biallelic single nucleotide polymorphism read frequencies for all 39 2013 PST field samples (RNA-seq). The mode for each distribution is given in parentheses. Additional file 5:
**All 2011 and 2012 PST-infected plant samples consisted of a single PST genotype.** Distribution of PST biallelic single nucleotide polymorphism read frequencies in RNA-seq datasets generated from wheat independently infected with PST isolates from 2011 and 2012. The mode for each distribution is given in parentheses. Additional file 6:
**PST 2013 field isolates can be partitioned into four distinct population clusters based on their genotypes.** **(a)** Application of the discriminant analysis of principal components (DAPC) multivariate method to 34,764 biallelic SNP sites defined the population structure and identified four groups of genetically related PST isolates. **(b)** Bayesian-based clustering of the full 34,806 synonymous SNP sites using the program STRUCTURE classified the PST isolates into four population clusters, which differed in partitioning of two isolates (PST-13/120 and PST-13/27) when compared with the DAPC assignment. Additional file 7:
**Details of analysis to (1) develop cluster-specific SNP markers to differentiate the four PST lineages, and (2) assess the influence of variation in gene expression on calculations of nucleotide diversity. **Text file. Additional file 8:
**Variation in expression profiles between members of a single PST population cluster are minimal.** **(a)** Of approximately 1,500 to 2,000 highly expressed genes within each population cluster, >99.7% were expressed by all members of the cluster. **(b)** Variation between members of a population cluster in expression levels for individual genes was low and comparable between clusters. Additional file 9:
**Differential expression analysis reveals population cluster-specific expression profiles.** **(a)** There were 49 to 254 genes significantly down-regulated and 122 to 240 genes significantly up-regulated for all isolates within a particular population cluster. Differential expression analysis was undertaken using Fisher’s exact test from the edgeR package to identify genes that were significantly differentially regulated between the four population clusters (false discovery rate <0.05; *P*-value <0.05). **(b)** Of the significantly up- or down-regulated genes, between 8.5 and 45.9% could be annotated with potential structural or enzymatic functions based on sequence similarity searches. **(c)** The effector candidate PST130_08031 was significantly down-regulated by isolates of cluster III. Additional file 10:
**SNP markers developed from the field pathogenomics data could differentiate the four emergent PST lineages.** Of the fifteen KASP assays developed, 11 could be used to differentiate particular lineages within the emergent PST population in the UK. Each closed circle represents the genotype of a single PST field sample. Blue circles, X:X; green circles, X:Y; red circles, Y:Y; open circles, H_2_0 negative control; grey circles, not determined. Where shown, background color reflects grouping of field samples within particular population clusters: cluster 1 = pink; cluster 2 = green; cluster 3 = blue; cluster 4 = red. Numbers reflect population clusters within each genotype group.

## Abbreviations

bp: base pair
DAPC: discriminant analysis of principal components
PST: *Puccinia striiformis* Westend. f. sp. *tritici* Eriks
SNP: single nucleotide polymorphism
