# Supplementary material for: Field pathogenomics reveals the emergence of a diverse wheat yellow rust population
Source: Genome Biol. 2015 Feb 25;16(1):23. doi: 10.1186/s13059-015-0590-8 (PMC4342793; doi:10.1186/s13059-015-0590-8)
Supplement: Additional file 7: — Details of analysis to (1) develop cluster-specific SNP markers to differentiate the four PST lineages, and (2) assess the influence of variation in gene expression on calculations of nucleotide diversity. Text file. [file 13059_2015_590_MOESM7_ESM.docx]

**(1) Population cluster-specific SNPs were shown to differentiate the four PST lineages using PCR-based assays**

We developed Kompetitive Allele Specific PCR (KASP) assays to assess the use of SNP-based markers for genotyping of pathogen-infected leaf samples. Fifteen SNPs that could either distinguish a specific cluster, or differentiate between each of two clusters, were used to design KASP assays. These markers were run on cDNA from nine PST field isolates representing the four clusters (Additional file 1: Table S6). Eleven assays successfully differentiated the particular cluster(s) within the 2013 PST population as expected based on the field transcriptomics data (Additional file 10). The remaining four assays showed polymorphism between samples but were not cluster-specific. These results suggest that cluster-specific SNPs can be identified and converted into PCR-based assays for future surveillance work.

**(2) Nucleotide diversity was not influenced by variation in gene expression between members of a population cluster**

Variation in gene expression between members of a population cluster could influence the calculation of nucleotide diversity. To assess this, we analyzed the expression patterns for approximately 1,500 to 2,000 highly expressed genes within each population cluster. For each cluster > 99.7% of these genes were expressed by all members of the cluster (Additional file 8A). Furthermore, expression level variation between members of a population cluster was low and comparable between clusters (Additional file 8B). Therefore, disparity in expression profiles between members of a single population cluster is unlikely to substantially influence the calculation of genetic diversity.
